# Anaerobic Fungi Isolated From Bactrian Camel Rumen Contents Have Strong Lignocellulosic Bioconversion Potential

**DOI:** 10.3389/fmicb.2022.888964

**Published:** 2022-07-19

**Authors:** Yihan Xue, Rui Shen, Yuqi Li, Zhanying Sun, Xiaoni Sun, Fengming Li, Xiaobin Li, Yanfen Cheng, Weiyun Zhu

**Affiliations:** ^1^Laboratory of Gastrointestinal Microbiology, National Center for International Research on Animal Gut Nutrition, Nanjing Agricultural University, Nanjing, China; ^2^College of Animal Science, Xinjiang Agricultural University, Ürümqi, China

**Keywords:** anaerobic fungi, bioconversion, strain isolation, hydrogenosome metabolism, lignocellulose degradation

## Abstract

This study aims to obtain anaerobic fungi from the rumen and fecal samples and investigates their potential for lignocellulosic bioconversion. Multiple anaerobic strains were isolated from rumen contents (CR1–CR21) and fecal samples (CF1–CF10) of Bactrian camel using the Hungate roll tube technique. After screening for fiber degradability, strains from rumen contents (*Oontomyces* sp. CR2) and feces (*Piromyces* sp. CF9) were compared with *Pecoramyces* sp. F1 (earlier isolated from goat rumen, having high CAZymes of GHs) for various fermentation and digestion parameters. The cultures were fermented with different substrates (reed, alfalfa stalk, *Broussonetia papyrifera* leaves, and *Melilotus officinalis*) at 39°C for 96 h. The *Oontomyces* sp. CR2 had the highest total gas and hydrogen production from most substrates in the *in vitro* rumen fermentation system and also had the highest digestion of dry matter, neutral detergent fiber, acid detergent fiber, and cellulose present in most substrates used. The isolated strains provided higher amounts of metabolites such as lactate, formate, acetate, and ethanol in the *in vitro* rumen fermentation system for use in various industrial applications. The results illustrated that anaerobic fungi isolated from Bactrian camel rumen contents (*Oontomyces* sp. CR2) have the highest lignocellulosic bioconversion potential, suggesting that the Bactrian camel rumen could be a good source for the isolation of anaerobic fungi for industrial applications.

## Introduction

Lignocellulosic biomass refers to residues from agricultural and forestry industries, which are plentiful renewable resources with an annual yield of about 10 billion tons (Nguyen et al., 2019). Recent research has shown that lignocellulosic biomass is a large reserve of carbon, and it could be used as a substrate for bioenergy production (Yamamoto et al., 2014; Nanda et al., 2015; Sawatdeenarunat et al., 2015). However, considerable amount of lignocellulosic biomass is simply discarded or burnt in the field, leading to resource wastage and environmental pollution (Liang et al., 2021). Therefore, it has become increasingly important and urgent to rationally utilize lignocellulosic biomass. The complex chemical composition and structure (which includes cellulose, hemicellulose, and lignin, interlinked with each other) of lignocellulosic biomass limit its utilization as animal feed or as a substrate for biofuel production. High energy and corrosive chemicals are needed to break lignocellulosic biomass (Zabed et al., 2016).

Recently, some methods have achieved substantial degradation of lignocellulose. These methods use physical and/or chemical processes. Protein engineering has also been used to improve the performance of the existing lignocellulose-degrading enzymes (Taherzadeh and Karimi, 2008; Singh et al., 2015; Shi et al., 2019). Although these pretreatment methods have been used to enhance the efficiency of hydrolysis of lignocellulosic biomass, their use inevitably brings about secondary pollution and increased cost (Abraham et al., 2020; Mankar et al., 2021). Currently, the bioconversion strategy is regarded as the most efficient and environmental-friendly approach for the industrial utilization of lignocellulosic biomass. This strategy utilizes carbohydrate-active enzymes secreted by microorganisms to break down stubborn structural polymers into easily metabolized monosaccharides that are subsequently converted into products (Balan, 2014). Research on improving the bioconversion efficiency of lignocellulose has received increased attention in recent years.

Anaerobic fungi, belonging to the phylum Neocallimastigomycota, are few in number in the rumen but they perform one of the main functions of fiber degradation in ruminants (Trinci et al., 1994; Hess et al., 2020). Anaerobic fungi secrete comprehensive fiber-degrading enzymes (Solomon et al., 2016). Previous studies have shown that anaerobic fungi have high lignocellulosic bioconversion potential (Saye et al., 2021; Kazemi Shariat Panahi et al., 2022; Li et al., 2022). During the anaerobic digestion, the main metabolites formed by anaerobic fungi are formate, acetate, ethanol, lactate, hydrogen, and carbon dioxide (Boxma et al., 2004; Wilken et al., 2021). High fiber degradability and organic acid production are the advantages of using anaerobic fungi as inoculants for the bio-fermentation of feeds. The anaerobic fungi led to the breakdown of plant fibers, and an acidic environment during fermentation favors the growth of other microorganisms (Sugiharto et al., 2016; He et al., 2020). In addition, hydrogen and ethanol, as the main metabolites of anaerobic fungi, can be used for the development of clean energy (Cantarel et al., 2009). Although anaerobic fungi have a low ethanol production capacity, multiple techniques for increasing ethanol production and using anaerobic fungi have been reported (Stevenson and Weimer, 2002; Ali et al., 2013; Kazemi Shariat Panahi et al., 2022). The potential use of anaerobic fungi for the production of bioethanol and biohydrogen is worth exploring. Apart from this, other metabolites such as acetate and formate formed during the fermentation are suitable substrates for additional downstream industrial processes (Saye et al., 2021). Therefore, harnessing the bioconversion potential of anaerobic fungi to process lignocellulosic biomass is of great value.

Advancements in sequencing technology and morphological techniques over the past decade have led to the description of a total of 20 genera of anaerobic fungi from a few sources (Joshi et al., 2018; Hanafy et al., 2020a,^2021^; Stabel et al., 2020; Li Y. Q. et al., 2021). Previous research has shown differences in the extent of lignocellulose degradation among anaerobic fungi of different genera (Gruninger et al., 2014; Hanafy et al., 2020b; Li Y. Q. et al., 2021). Although studies have shown that the fungal community composition is similar in different sections of the ruminants’ gut (Davies et al., 1993a; Rabee et al., 2019), there are huge differences in their functions, and also their community structure and functions differ between samples of rumen and feces (Agustina et al., 2022). Therefore, we hypothesized that there were also significant differences between anaerobic fungi isolated from different rumen contents and feces.

In this study, a number of strains were isolated from rumen contents and fecal samples of Bactrian camel and subsequently compared with *Pecoramyces* sp. F1 (isolated from goat rumen liquid, having high glycoside hydrolases) (Jin et al., 2011) for lignocellulosic bioconversion using different substrates. The substrates included reed, alfalfa stalk, *Broussonetia papyrifera* leaves, and *Melilotus officinalis*, which have different lignocellulose contents. We expected differences in hydrogen production, lignocellulose degradation efficiency, and fermentation metabolite production to assess our hypothesis.

## Materials and Methods

### Source of Inoculum and Isolation Procedures

The complex medium used for isolation was according to the method of Cheng et al. (2009), which contained 1% wt/vol rice straw (hereafter listed as 1% rice straw) and antibiotics (1,600 IU/ml penicillin; 2,000 IU/ml streptomycin). The rice straw used was rinsed three times with distilled water, dried at 65°C in a blast drying oven (DHG-9245A, Shanghai Youyi Instrument Co., Ltd., Shanghai, China), pulverized, passed through a 3 mm sieve, sealed, and then stored at room temperature. Every 1,000 ml of the complex medium contained 150 ml of buffer solution A, 150 ml of buffer solution B, 150 ml of buffer solution C, 400 ml of basal medium (which contains 6 g NaHCO_3_, 10 g tryptone, and 2.5 g yeast extract), 150 ml of cell-free rumen liquid (the fresh rumen fluid centrifuged at 12,000 *g* for 5 min and the supernatant collected and stored at −20°C till use), 1 g of L-cysteine hydrochloride, and 1 ml of 0.1% (wt/vol) resazurin. Buffer solution A contained 0.3 g of K_2_HPO_4_ per 100 ml; buffer solution B contained 0.3 g of KH_2_PO4, 0.6 g of (NH_4_)_2_SO_4_, and 0.06 g of MgSO_4_⋅7H_2_O; and buffer solution C contained 0.6 g of NaCl and 0.06 g of CaCl_2_⋅2H_2_O per 100 ml. The complex medium (90 ml portions) was dispensed into 180 ml serum bottles under continuous flow of CO_2_. Thereafter, the serum bottles were sterilized by autoclaving at 121°C for 20 min.

Fresh rumen contents (5 g) and fresh feces (5 g) were collected from a Bactrian camel in Jimunai County (latitude 47°00′–47°59′ N and longitude 85°33′–87°09′ E), Xinjiang, China. After preheating 90 ml of the complex medium to be inoculated at 39°C for 2 h in advance, remove the aluminum cap and rubber stopper, and quickly transfer the collected samples to the complex medium. The cultures were incubated at 39°C in an incubator (HGPN-11-163, HENGZI, Shanghai, China) and transferred every 3 days. After three consecutive batch culture series, fungal growth (marked by rice straw floating in the complex medium) and methane production [detected by gas chromatography (GC)] were checked to ensure that mixed cultures of anaerobic fungi and methanogens were obtained (Cheng et al., 2009). An aliquot of 1 ml culture was inoculated into 9 ml anaerobic solution (complex medium without rumen liquid and basal medium) and diluted two times, and then 0.5 ml of the diluted culture was inoculated into 10 ml of the cellobiose medium supplemented with 2% agarose in the Hungate roll tube (Jin et al., 2011). After single fungal colonies were formed (in 3 days), they were picked up and transferred into the fresh complex medium with 1% rice straw (Ozkose et al., 2001). This process was repeated up to three times, until the fungal colonies appeared uniform under a microscope. After isolation, the cultures were maintained at 39°C in the complex medium with 1% rice straw and transferred every 3 days.

### Identification of Anaerobic Fungi

For the genetic characterization, biomass was collected from 10 ml of 3-day cultures after centrifugation at 12,000 *g* for 10 min. DNA was extracted from the precipitate using DNeasy PowerPlant Pro Kit (Qiagen, Germantown, Maryland) according to the manufacturer’s instructions. The region encompassing ITS1, 5.8S, ITS2, and D1/D2 of the LSU rRNA was targeted for amplification using the primers ITS5 (5′-GGAAGTAAAAGTCGTAACAAGG-3′) and NL4 (5′-TCAACATCCTAAGCGTAGGTA-3′) (Dagar et al., 2011). The PCR protocol consisted of an initial denaturation for 5 min at 95°C followed by 40 cycles of denaturation at 95°C for 1 min, annealing at 55°C for 1 min, elongation at 72°C for 2 min, and a final extension of 72°C for 20 min (Dagar et al., 2011). The PCR products were Sanger sequenced at the Tsingke Biotechnology Co., Ltd., Nanjing, China. All raw sequences were deposited in the NCBI GenBank database under the accession number ON514405-ON514425, ON51427-ON514437.

The raw sequences were compared with the sequences available in the BLAST GenBank.^1^ The default settings for the neighbor-joining method in MEGA X were applied to construct the phylogenetic tree (Kumar et al., 2018).

### Comparison of Isolated Anaerobic Fungi

#### Screening of Efficient Fiber Degrading Anaerobic Fungi

A 10 ml portion of 3-day-old cultures, isolated from the camel rumen contents or feces, was inoculated into 90 ml fresh complex medium and incubated at 39°C for 96 h without shaking, with 1% of the rice straw (milled to pass through a 3 mm sieve) as a substrate. Each group had 4 replicates and a blank control to correct for the gas production. At 12, 24, 32, 40, 48, 60, 72, and 96 h of fermentation, the head-space gas was measured using the pressure transducer technique (PTT) and then vented to return the pressure back to ambient condition (Theodorou et al., 1994). At 96 h, pH was measured immediately after removing crimp seals and stoppers. The substrate after the fermentation was washed with deionized water and dried at 105°C in a blast drying oven (DHG-9245A, Shanghai Youyi Instrument Co., Ltd., Shanghai, China) to calculate dry matter digestibility (DMD). Anaerobic fungi strains that had the highest DMD and total gas production were taken to the next experiment. Two fungi (one each from the rumen contents and feces) were considered as having efficient dry matter degrading ability. Taxonomic features of these two fungi (in the medium with 1% rice straw) were examined by microscopy (DMi8, Leica, Germany) (Li Y. Q. et al., 2021).

#### Carbon Source Usage and Sample Collection

Two strains isolated from the Bactrian camel rumen contents or feces, which had the highest DMD and *Pecoramyces* sp. F1. (Li et al., 2017), were inoculated separately into 90 ml media with several 1% wt/vol substrates (e.g., reed, alfalfa stalk, *B. papyrifera* leaves, and *M. officinalis*). The substrates were milled to pass through a 3 mm sieve before incubation. The experiment was set up with 12 different treatment groups (3 anaerobic fungi × 4 substrates), 4 replicates in each group, and a blank control to correct for gas production. The incubation was done at 39°C for 96 h without shaking. At 12, 24, 32, 40, 48, 60, 72, and 96 h of the fermentation, the head-space gas was measured using the PTT and then vented to return the pressure back to ambient condition. An airbag was connected to collect the gas for the determination of composition (Theodorou et al., 1994). Aliquots of 4 ml supernatant of culture were collected and stored at −20°C for the analysis of ethanol, formate, acetate, and lactate. In addition to this, for the analysis of two main fiber-degrading enzyme activities, aliquots of 2 ml supernatant of the culture were collected and stored in liquid nitrogen. The remaining fermentation medium was centrifuged at 12,000 *g* for 20 min to collect the residue, which was washed with deionized water and dried at 105°C in a blast drying oven to calculate DMD. Thereafter, it was used for the determination of the neutral detergent fiber (NDF), acid detergent fiber (ADF), neutral detergent solutes (NDS), cellulose, hemicellulose, and lignin, according to the methods reported in Zhu et al. (2001).

### Determination of the Composition of Gas

The relative abundance of hydrogen in the head-space gas in the bottle was measured by a GC system (Agilent 7890B, Agilent, California, United States). The gas sample was injected into a GC, and the gases were separated on packed GC columns (Fused Silica Capillary Column, Supelco, United States) at 80°C column temperature, 200°C injection temperature, 200°C TCD temperature, and air pressure and carrier gas (nitrogen) pressure of 0.05 MPa. The volume of hydrogen was calculated according to the method of Li et al. (2020).

### Analysis of Fermentation Metabolites and Fiber-Degrading Enzyme Activity

The pH was measured at the end of fermentation immediately after removing crimp seals and stoppers from the serum vials (Seven2GO S2, METTLER TOLEDO, Switzerland). For the analyses of ethanol and acetate, 1 ml of supernatant was added to 1.5 ml centrifuge tubes with added 0.2 ml deproteinized metaphosphate solution containing crotonic acid. After centrifugation at 12,000 *g* for 10 min, the supernatant was filtered through a filter membrane (size 0.22 μm, SCAA-102; ANPEL, Shanghai, China); 1 μl of the filtered liquid was analyzed by GC (Agilent 7890B, Agilent, California, United States) at 40°C column temperature, 220°C injection temperature, and 230°C TCD temperature. The pressures of air, N_2_ carrier gas, and H_2_ were 0.05 MPa, according to the method of Li et al. (2016). Formate was measured by the Formate Assay Kit (K-FORM, Megazyme, Ireland), and lactate was measured with the Lactate Assay Kit (A019-2, Nanjing Jiancheng Biotechnology Institute, China) according to the manufacturer’s instructions (Zhu et al., 2001).

The activities of carboxymethyl cellulase (CMCase), a cellulose-degrading enzyme, and of xylanase, a hemicellulose-degrading enzyme, were measured according to the method of Li et al. (2020). One unit of CMCase and xylanase activity was defined as 1 μmol of glucose released per ml of supernatant per minute (U/ml/min) and as 1 μmol of xylose released per ml of supernatant per minute (U/ml/min), respectively.

### Analysis of the Chemical Composition of Different Substrates

A fiber analyzer (Ankom A200i; Ankom Technology, New York, United States) and a Muffle furnace (SX2-4-10N, Yiheng, Shanghai, China) were used for the determination of NDF, ADF, and lignin contents (Vansoest et al., 1991). Briefly, samples were treated in the neutral detergent solution in the fiber analyzer; the dissolved part was NDS, and the remaining part was NDF. The NDF was further treated using an acid detergent solution to dissolve hemicellulose and to obtain ADF. After treatment with 72% sulfuric acid, the ADF was digested to dissolve cellulose, and the residue, a mixture of lignin and silicate, was obtained. The residue was washed to remove lignin and to obtain silicate. The method of Niu et al. (2018) was used to calculate the contents of cell soluble or the soluble solutes in the neutral detergent solution, cellulose, and hemicellulose.

### Statistical Analysis

All experimental data were entered into Excel 2019 (Microsoft, Redmond, WA, United States) and preliminary processed, and then, Duncan’s analysis of one-way AVONA was used to calculate data at 95% level significance by using SPSS 26.0 (IBM SPSS Statistics, version 26.0; IBM Corp, Armonk, NY, United States). Data were presented as mean ± standard error of the mean.

## Results

### Characterization of Fungi From Isolation

**FIGURE 1:**
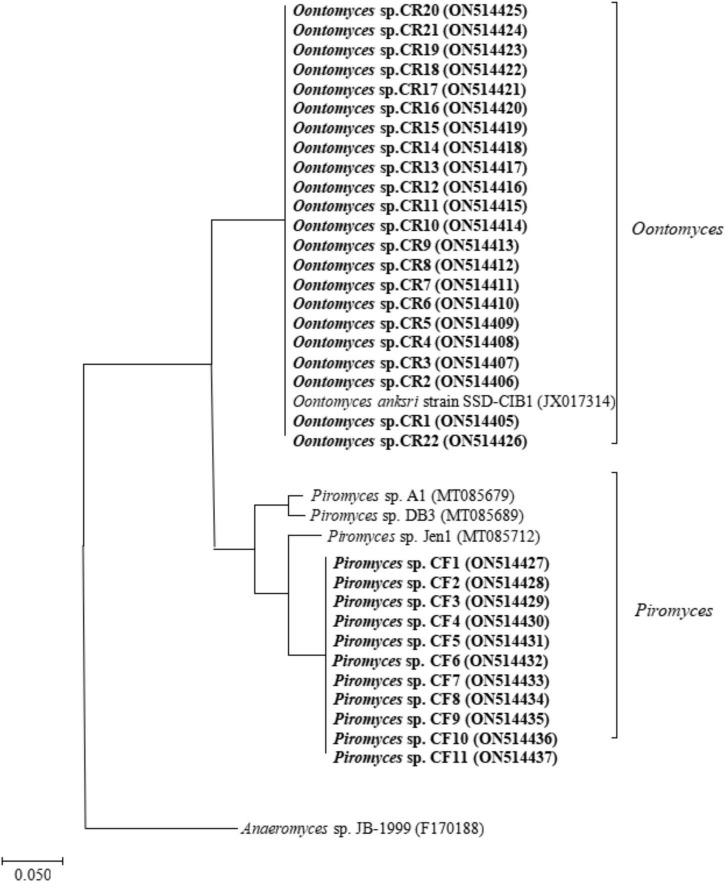
The phylogenetic tree of ITS1 sequences of anaerobic fungal strains isolated from the rumen and feces of Junggar Bactrian camels.

A total of 21 and 10 cultures were isolated from the Bactrian camel rumen contents (CR1–CR21) and feces (CF1–CF10), respectively. After PCR amplification, we obtained 31 PCR products and subsequently obtained the raw sequence. After comparing with sequences available, 31 isolates were collected and identified as described in Supplementary Table 1. The results of ITS sequencing showed that the rumen-isolated anaerobic fungal strains were *Oontomyces* sp., and the fecal-isolated anaerobic fungal strains were *Piromyces* sp. The phylogenetic tree showed that the similarity between the rumen-isolated anaerobic fungi *Oontomyces* sp. CR1–CR22 and *Oontomyces anksri* strains SSD-CIB1 belonged to the same clade, and the similarity between fecal-isolated anaerobic fungi *Piromyces* sp. CF1–CF11 and *Piromyces* sp. Jen1 belonged to the same clade (Figure 1).

After 96 h of fermentation, the total gas production, DMD, and pH are presented in Supplementary Tables 2, 3. Anaerobic fungus *Oontomyces* sp. CR2 had the highest total gas production, DMD, and gas production per DM among CR1–CR21, with relatively lower pH than the other isolates from the Bactrian camel rumen contents (*P* < 0.05). For the isolates from fecal samples, the anaerobic fungus *Piromyces* sp. CF9 had the highest total gas production, DM loss, and gas production per DM among CF1–CF10, with relatively lower pH than the others from the Bactrian camel feces (*P* < 0.05) (Supplementary Table 3). Thus, *Oontomyces* sp. CR2 and *Piromyces* sp. CF9 were considered as the anaerobic fungi with efficient dry matter degradation ability and were used in further experiments.

**FIGURE 2:**
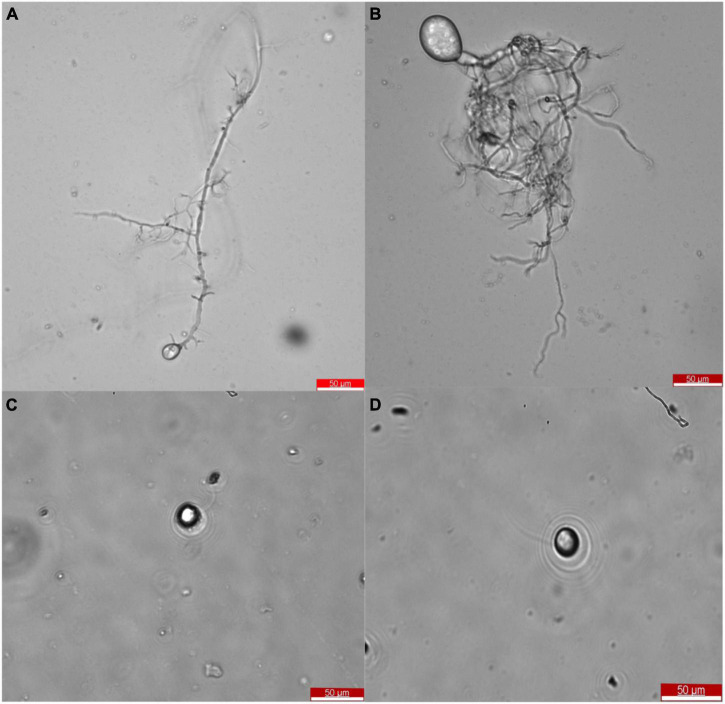
Morphology of *Oontomyces* sp. CR2 **(A,C)** and *Piromyces* sp. CF9 **(B,D)**. Scale bar indicates 50 μm **(A,B)**.

After the selection of efficient fiber degrading fungi, *Oontomyces* sp. CR2 and *Piromyces* sp. CF9, they were identified morphologically. The taxonomic features are shown in Figure 2. Morphological characteristics showed that these fungi with monocentric thallus have spherical or oval sporangium with filamentous rhizoids. Zoospores are uniflagellate and spherical.

### Gas and Hydrogen Production From Different Fermentation Substrates

**FIGURE 3:**
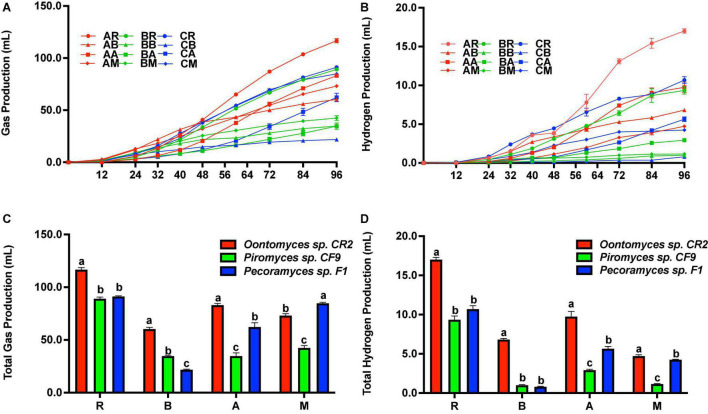
Cumulative gas and H_2_ production **(A,B)**, and total gas and H_2_ production **(C,D)** after 96 h fermentation with *Oontomyces* sp. CR2, *Piromyces* sp. CF9, and *Pecoramyces* sp. F1 using different substrates (R, Reed; B, *Broussonetia papyrifera* leaves; A, Alfalfa stalk; M, *Melilotus officinalis*). The error bars represent the standard error of the mean (*n* = 4), and means with different letters in the same column are different at *P* < 0.05.

Figure 3A shows the cumulative gas production. Before 48 h of fermentation, the highest cumulative gas production was with *B. papyrifera* leaves using *Oontomyces* sp. CR2 as the inoculum. But after 48 h to the end of the fermentation, the highest cumulative gas production was with reed as the substrate. At the end of fermentation, *Oontomyces* sp. CR2 had the highest total hydrogen production (Figure 3D) and gas production with reed, *B. papyrifera* leaves, and alfalfa stalk (*P* < 0.05) as substrates; however, *Pecoramyces* sp. F1 produced the highest gas with *M. officinalis* (*P* < 0.05) (Figure 3C).

The cumulative hydrogen production is shown in Figure 3B. Before 50 h of fermentation, the highest cumulative hydrogen production was with reed as a substrate on using *Pecoramyces* sp. F1 as the inoculum. From 50 h onward, the highest cumulative hydrogen production was with reed on using *Oontomyces* sp. CR2 as the inoculum. At the end of the fermentation, *Oontomyces* sp. CR2 produced the highest total hydrogen in every substrate evaluated (*P* < 0.05) (Figure 2D).

### Different Digestions of Chemical Composition Among Substrates by Fermentation

Different substrates have different chemical compositions (Table 1). Reed has the lowest NDS content (*P* < 0.05) and high lignin content. On the contrary, *B. papyrifera* leaves have the highest NDS content (*P* < 0.05) and the lowest NDF, ADF, cellulose, and lignin contents (*P* < 0.05). There is not much difference in composition between alfalfa stalk and *M. officinalis*.

**TABLE 1:** Chemical composition of reed, alfalfa stalk, *Broussonetia papyrifera* leaves, and *Melilotus officinalis*.

Substrate	Reed	*Broussonetis papyrifera* leaves	Alfalfa stalk	*Melilotus officinalis*
NDF (%)	80.09 ± 2.41^a^	32.09 ± 4.86^c^	69.73 ± 0.91^b^	71.84 ± 2.05^ab^
NDS (%)	19.91 ± 2.41^c^	67.91 ± 4.86^a^	30.27 ± 0.91^b^	28.17 ± 2.05^bc^
ADF (%)	56.44 ± 2.63^a^	16.40 ± 2.59^b^	54.32 ± 0.75^a^	52.91 ± 1.42^a^
Cellulose (%)	38.40 ± 1.20^a^	11.55 ± 1.45^b^	40.87 ± 0.74^a^	38.48 ± 0.45^a^
Hemicellulose (%)	23.64 ± 0.22^a^	15.69 ± 2.28^b^	15.42 ± 0.15^a^	18.93 ± 0.63^b^
Lignin (%)	17.68 ± 1.23^a^	4.14 ± 0.86^c^	12.64 ± 0.20^ab^	13.52 ± 0.94^ab^
*P*-value	<0.05	<0.05	<0.05	<0.05

*The error bars represent the standard error of the mean (n = 4), and means with different letters in the same column are different at P < 0.05.*

**FIGURE 4:**
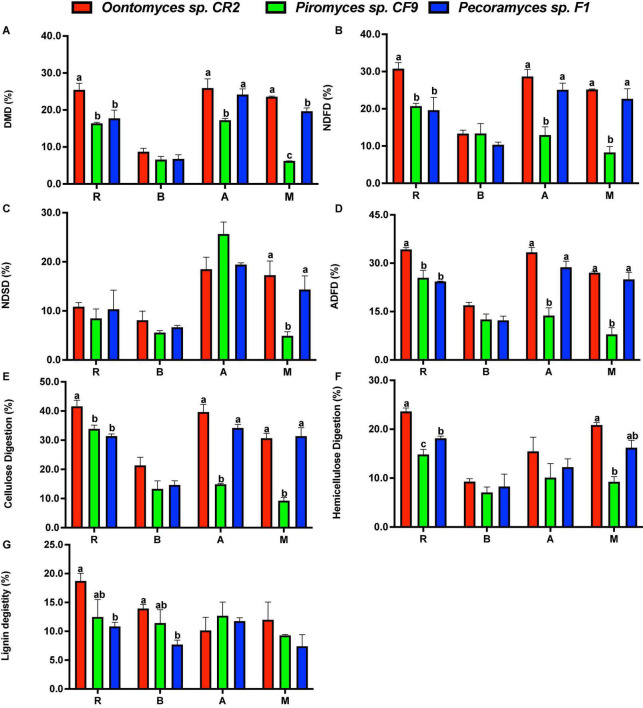
Degradation of reed, alfalfa stalk, *Broussonetia papyrifera* leaves, and *Melilotus officinalis* after fermentation. The figure includes DMD **(A)**, NDF digestion **(B)**, NDS digestion **(C)**, ADF digestion **(D)**, cellulose digestion **(E)**, hemicellulose digestion **(F)** and lignin digestion **(G)** (R, Reed; B, *Broussonetia papyrifera* leaves; A, Alfalfa stalk; M, *Melilotus officinalis*). The error bars represent the standard error of the mean (*n* = 4), and means with different letters in the same column are different at *P* < 0.05.

The extent of degradation of different substrates after the fermentation is shown in Figure 4. By comparing the degradation characteristics of different anaerobic fungi on lignocellulose substrates, high-degrading anaerobic fungi were screened, thus laying a foundation for further exploration of their application potential (Dagar et al., 2015a,^2018^). The fermentation using different fungi and substrates shows a certain pattern in this research. The highest DMD for all the substrates was by *Oontomyces* sp. CR2, while this fungus had the highest NDFD for all the substrates except *B. papyrifera* leaves (*P* < 0.05). For all the substrates, the highest ADFD, cellulose digestion, and hemicellulose digestion were using *Oontomyces* sp. CR2 (*P* < 0.05). With *Oontomyces* sp. CR2, the highest lignin digestion was for all the substrates except alfalfa stalk.

### Fermentation Metabolites and Fiber-Degrading Enzyme Activities of Cultures With Different Substrates

**FIGURE 5:**
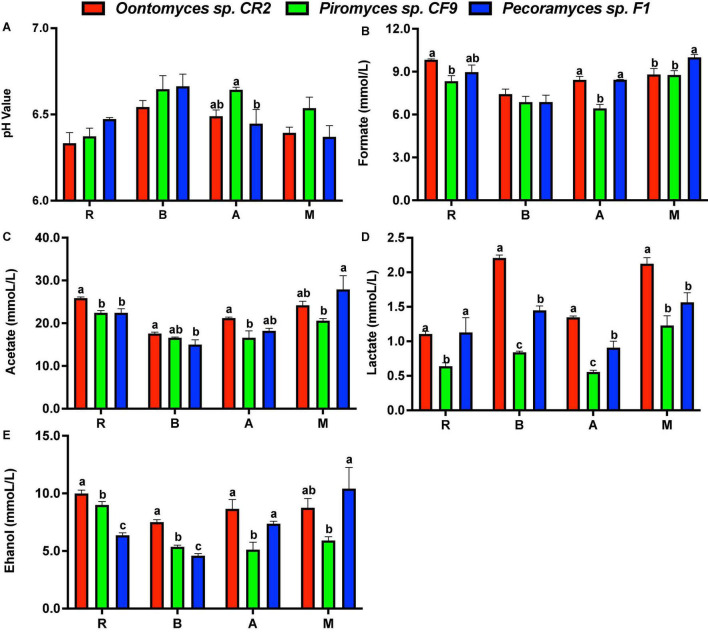
The pH value **(A)** and concentrations of formate **(B)**, acetate **(C)**, lactate **(D)**, and ethanol **(E)** in the supernatant of different fermentations (R, Reed; B, *Broussonetia papyrifera* leaves; A, Alfalfa stalk; M, *Melilotus officinalis*). The error bars represent the standard error of the mean (*n* = 4), and means with different letters in the same column are different at *P* < 0.05.

Using the *Pecoramyces* sp. F1 culture, the pH value drop was lower on using reed and *B. papyrifera* leaves as substrates than using *Oontomyces* sp. CR2 or *Piromyces* sp. CF9, while using alfalfa and *M. officinalis* as substrates, *Piromyces* sp. CF9 had the least pH value drop, followed by *Oontomyces* sp. CR2 and *Pecoramyces* sp. F1 (Figure 5).

Acetate was the main metabolite in the supernatant, followed by formate, ethanol, and lactate. Especially, *Oontomyces* sp. CR2 had the highest values of almost all metabolites. For example, the lactate content was highest except for reed (*P* < 0.05), and acetate and ethanol contents were highest except for *M. officinalis* (*P* < 0.05), while *Pecoramyces* sp. F1 had the highest formate content for *M. officinalis* (*P* < 0.05) (Figure 5).

The results of fiber-degrading enzyme activities are shown in Supplementary Figure 2. The results showed that *Pecoramyces* sp. F1, *Oontomyces* sp. CR2, and *Piromyces* sp. CF9 showed differences in enzyme activity. Former studies showed that different genera of anaerobic fungi exhibited significantly different enzyme activities, which were the same as our results (Dagar et al., 2018). For CMCase activity, *Pecoramyces* sp. F1 had higher activity than others for reed, alfalfa stalk, and *M. officinalis*, but *Oontomyces* sp. CR2 had the highest CMCase activity for *B. papyrifera* leaves (*P* < 0.05). For xylanase activity, *Pecoramyces* sp. F1 had the highest activity in every substrate.

## Discussion

### Higher Number of Anaerobic Fungi Isolated From the Bactrian Camel Rumen Contents Than Feces

The numbers of strains isolated from rumen were much higher than feces. Anaerobic fungi exist in all parts of the digestive tract, but due to limitations in accessing locations for sampling, anaerobic fungi have been isolated from rumen and feces in most cases (Voigt et al., 2021). The unique anaerobic environment of the rumen and suitable pH promote the growth of anaerobic fungi and make them degrade the feed that stays in the rumen for a long duration (Gruninger et al., 2014; Li M. M. et al., 2021). Previous studies have reported that almost 90% of the fungal population inhabited the rumen, illustrating that the total population of anaerobic fungi in the rumen is considerably larger than that in the omasum and hindgut organs (Davies et al., 1993b; Mao et al., 2016; Edwards et al., 2017). These results demonstrate that anaerobic fungi are isolated easily and in more numbers from the rumen, due to their abundance in this part of the digestive tract.

### Anaerobic Fungus Isolated From the Bactrian Camel Rumen Contents Produced Higher Hydrogen

The isolated anaerobic fungus from the rumen contents, *Oontomyces* sp. CR2 had the highest total hydrogen production in every substrate (*P* < 0.05). Due to the absence of mitochondria in anaerobic fungi, the hydrogenosome is used to produce ATP (Borneman et al., 1989; Baykara, 2018). The hydrogenosomes produce hydrogen by using protons as electron acceptors during mixed-acid fermentation of monomeric sugars (predominantly glucose and xylose) derived from cellulose and hemicellulose (Saye et al., 2021). In the rumen environment, anaerobic fungi come into contact with a large number of substrates that have high cellulose and hemicellulose contents, which act as substrates for the production of hydrogen and an array of metabolites. The rumen is a relatively closed chamber having vast kinds of microorganisms. Due to the presence of methanogens, the accumulation of hydrogen is limited because it gets converted to methane (Hungate et al., 1970). Mizrahi et al. (2021) demonstrated that methanogenesis is likely to facilitate the activity of hydrogenase and the generation of hydrogen and ATP by anaerobic fungi (Hackstein et al., 2019). Anaerobic fungi in the rumen are exposed to higher concentrations of methane than anaerobic fungi in feces, which also explains higher hydrogen production by isolates from rumen contents. The rumen environment promotes the production of metabolites of hydrogenosome in anaerobic fungi, and this feature is preserved even in the anaerobic fungi isolated from the *in vitro* rumen fermentation system (Ma et al., 2022). In our study, although fungi from different sources share the same fermentation conditions (temperature and substrate types) in the *in vitro* system, strains isolated from rumen contents had higher hydrogen production than those from feces. These results further support the advantages of hydrogen metabolism in anaerobic fungi isolated from rumen contents. Also, feces get exposed to oxygen, and this could be the reason for lower abundance and activity of fungi isolated from feces.

### Anaerobic Fungus Isolated From the Bactrian Camel Rumen Contents Is More Suitable as a Bio-Fermented Feed Inoculant

*Oontomyces* sp. CR2 had the highest digestibility for most components of different substrates. Recently, Hanafy et al. (2020b) proposed the existence of a strong anaerobic fungi genus–host preference, which for some genera are encountered only in a specific animal. The anaerobic fungus, *Oontomyces anksri* gen.nov., sp. nov., was first isolated from the forestomach of the Indian camel (*Camelus dromedarius*) (Dagar et al., 2015b). Camel’s unique physical characteristics make it specially adapted to arid environments, and it has developed physiological mechanisms to face heat stress, dehydration, and shortage of nutrients (Guerouali and Wardeh, 1998). Meanwhile, the camel has a distinctive gastrointestinal morphology and is often described as a pseudoruminant. Its highly enlarged foregut comprises three distinct regions (Wilson, 1989). Due to the unique digestive system, the retention time of feed particles in camel is higher as compared with that in sheep and cattle, which increases the exposure of plant biomass to symbiotic microorganisms and helps in the efficient digestion of low-quality fibrous diets (Samsudin et al., 2011). The highest digestibility of *Oontomyces* sp. CR2, among the three anaerobic fungi investigated in our study, could be attributed to the unique digestive system and its unique environment. When anaerobic fungi with high capability to digest are used as an inoculant, the composition of the feed changes dramatically during fermentation. The plant cell wall of roughages gets degraded (de Vries and Visser, 2001), the contents of anti-nutritional factors are decreased (Abd El-Hack et al., 2018), and the palatability is improved (Yang et al., 2001; Oladosu et al., 2016). Our results showed that the anaerobic fungus isolated from the Bactrian camel rumen contents is a promising candidate for use as a feed inoculant in a bio-fermenter.

### Anaerobic Fungus Isolated From the Bactrian Camel Rumen Contents Has Higher Potential for Industrial Applications

In addition to gas metabolites (hydrogen and carbon dioxide), anaerobic fungi also secrete a range of organic acids in their fermentation pathways (Breton et al., 1989; Boxma et al., 2004). These compounds have the potential for use as valuable substrates in various industrial processes. These substrates could have higher economic value than biogas as the final product (Silva et al., 2021). In our study, the fermentation products mainly included formate, acetate, lactate, and ethanol, which are consistent with the previous reports, and the different accumulation of metabolites can result in a variety of pH values (Li Y. Q. et al., 2021).

Moreover, *Oontomyces* sp. CR2, an anaerobic fungus isolated from the Bactrian camel rumen contents, showed significant differences in the production of a number of metabolites. Earlier research has shown that when the same anaerobic fungus is used to ferment different substrates, the types of metabolites do not change, but their proportions and contents change (Borneman et al., 1989). In the pathway of glucose metabolism, anaerobic fungi convert glucose to pyruvate in the cytosol or to malate and further to pyruvate in the hydrogenosome (Akhmanova et al., 1999; Li et al., 2016). After entering the hydrogenosome, malate is converted to CO_2_ and H_2_ or to pyruvate and further to format and acetate (Akhmanova et al., 1999). The higher levels of metabolite produced by *Oontomyces* sp. CR2 (isolated from the Bactrian camel rumen contents) may be attributed to two reasons. First, although different isolated anaerobic fungi fermented all the substrates in our study, *Oontomyces* sp. CR2 led to the degradation of higher proportion of the substrate to monosaccharides, which served as available substrates to facilitate the production of higher levels of metabolites. Second, higher levels of metabolite production created a lower pH acidic environment, which is more suitable for the growth of anaerobic fungi. This enhanced the metabolic capacity of cytosol and hydrogenosome (Saye et al., 2021).

## Conclusion

Based on the observed high dry matter digestion and production of hydrogen and other fermentation metabolites, the anaerobic fungus (*Oontomyces* sp. CR2) isolated from the Bactrian camel rumen contents has the highest lignocellulosic bio-convention potential. The number of anaerobic fungi isolated from rumen contents was higher than that from feces, and the fungi isolated from the rumen contents had higher dry matter digestion and higher potential for various industrial applications. These results further highlight the advantages the rumen contents offer in the isolation of anaerobic fungi. The use of anaerobic fungi, isolated from the rumen, as an inoculum for various bio-conversion applications could be a good strategy for the future.

We used a single-substrate approach for the isolation of anaerobic fungi, which might have led to the isolation of fewer genera of anaerobic fungi. It would be interesting to use multi-substrates for the isolation. This might increase the number of genera of isolated anaerobic fungi having promising traits. Studies on these lines are suggested.

## Data Availability Statement

The datasets presented in this study can be found in online repositories. The names of the repository/repositories and accession number(s) can be found in the article/Supplementary Material.

## Author Contributions

YX, YL, and RS conducted the experiment and analyzed the data. YX, XS, and ZS conceived and designed the work. YX, FL, XL, YC, and WZ wrote and revised the article. All authors contributed to the article and approved the submitted version.

## Conflict of Interest

The authors declare that the research was conducted in the absence of any commercial or financial relationships that could be construed as a potential conflict of interest.

## Publisher’s Note

All claims expressed in this article are solely those of the authors and do not necessarily represent those of their affiliated organizations, or those of the publisher, the editors and the reviewers. Any product that may be evaluated in this article, or claim that may be made by its manufacturer, is not guaranteed or endorsed by the publisher.

## Footnotes

1
http://www.ncbi.nlm.nih.gov/BLAST/Blast.cgi

## References

[B1] Abd El-Hack M. E. Samak D. H. Noreldin A. E. Arif M. Yaqoob H. S. Swelum A. A. (2018). Towards saving freshwater: halophytes as unconventional feedstuffs in livestock feed: a review. *Environ. Sci. Pollut. Res.* 25 14397–14406. 10.1007/s11356-018-2052-9 29700747

[B2] Abraham A. Mathew A. K. Park H. Choi O. Sindhu R. Parameswaran B. (2020). Pretreatment strategies for enhanced biogas production from lignocellulosic biomass. *Bioresou. Technol.* 301:122725. 10.1016/j.biortech.2019.122725 31958690

[B3] Agustina S. Wiryawan K. G. Suharti S. Meryandini A. (2022). The enrichment process and morphological identification of anaerobic fungi isolated from buffalo rumen. *Biodiversitas J. Biol. Diversity* 23 469–477.

[B4] Akhmanova A. Voncken F. G. Hosea K. M. Harhangi H. Keltjens J. T. Op Den Camp H. J. (1999). A hydrogenosome with pyruvate formate-lyase: anaerobic chytrid fungi use an alternative route for pyruvate catabolism. *Mole. Microbiol.* 32 1103–1114. 10.1046/j.1365-2958.1999.01434.x 10361311

[B5] Ali S. S. Nugent B. Mullins E. Doohan F. M. (2013). Insights from the fungus fusarium oxysporum point to high affinity glucose transporters as targets for enhancing ethanol production from lignocellulose. *Plos One* 8:e54701. 10.1371/journal.pone.0054701 23382943 PMC3559794

[B6] Balan V. (2014). Current challenges in commercially producing biofuels from lignocellulosic biomass. *ISRN Biotechnol.* 2014:463074. 10.1155/2014/463074 25937989 PMC4393053

[B7] Baykara S. Z. (2018). Hydrogen: a brief overview on its sources, production and environmental impact. *Int. J. Hydrogen Energy* 43 10605–10614.

[B8] Borneman W. S. Akin D. E. Ljungdahl L. G. (1989). Fermentation products and plant-cell wall-degrading enzymes produced by monocentric and polycentric anaerobic ruminal fungi. *Appl. Environ. Microbiol.* 55 1066–1073. 10.1128/Aem.55.5.1066-1073.1989 2757372 PMC184255

[B9] Boxma B. Voncken F. Jannink S. van Alen T. Akhmanova A. van Weelden S. W. H. (2004). The anaerobic chytridiomycete fungus *Piromyces* sp E2 produces ethanol via pyruvate : formate lyase and an alcohol dehydrogenase E. *Mole. Microbiol.* 51 1389–1399. 10.1046/j.1365-2958.2003.03912.x 14982632

[B71] Breton A. Bernalier A. Bonnemoy F. Fonty G. Gaillard B. Gouet P. (1989). Morphological and metabolic characterization of a new species of strictly anaerobic rumen fungus – *Neocallimastix-joyonii*. *FEMS Microbiol. Lett.* 58, 309–314. 10.1111/j.1574-6968.1989.tb03065.x 2744422

[B10] Cantarel B. L. Coutinho P. M. Rancurel C. Bernard T. Lombard V. Henrissat B. (2009). The Carbohydrate-Active EnZymes database (CAZy): an expert resource for glycogenomics. *Nucleic Acids Res.* 37 D233–D238. 10.1093/nar/gkn663 18838391 PMC2686590

[B11] Cheng Y. F. Edwards J. E. Allison G. G. Zhu W. Y. Theodorou M. K. (2009). Diversity and activity of enriched ruminal cultures of anaerobic fungi and methanogens grown together on lignocellulose in consecutive batch culture. *Bioresou. Technol.* 100 4821–4828. 10.1016/j.biortech.2009.04.031 19467591

[B12] Dagar S. S. Kumar S. Griffith G. W. Edwards J. E. Callaghan T. M. Singh R. (2015a). A new anaerobic fungus (*Oontomyces anksri* gen. nov., sp. nov.) from the digestive tract of the Indian camel (*Camelus dromedarius*). *Fungal Biol.* 119 731–737. 10.1016/j.funbio.2015.04.005 26228561

[B13] Dagar S. S. Kumar S. Mudgil P. Puniya A. K. (2018). Comparative evaluation of lignocellulolytic activities of filamentous cultures of monocentric and polycentric anaerobic fungi. *Anaerobe* 50 76–79. 10.1016/j.anaerobe.2018.02.004 29454109

[B14] Dagar S. S. Kumar S. Mudgil P. Singh R. Puniya A. K. (2011). D1/D2 domain of large-subunit ribosomal DNA for differentiation of *Orpinomyces* spp. *Appl. Environ. Microbiol.* 77 6722–6725. 10.1128/AEM.05441-11 21784906 PMC3187145

[B15] Dagar S. S. Singh N. Goel N. Kumar S. Puniya A. K. (2015b). Role of anaerobic fungi in wheat straw degradation and effects of plant feed additives on rumen fermentation parameters *in vitro*. *Benefical Microbes* 6 353–360. 10.3920/BM2014.0071 25391347

[B16] Davies D. R. Theodorou M. K. Brooks A. E. Trinci A. P. (1993a). Influence of drying on the survival of anaerobic fungi in rumen digesta and faeces of cattle. *FEMS Microbiol. Lett.* 106 59–63. 10.1111/j.1574-6968.1993.tb05935.x 8440466

[B17] Davies D. R. Theodorou M. K. Lawrence M. I. Trinci A. P. (1993b). Distribution of anaerobic fungi in the digestive tract of cattle and their survival in faeces. *Microbiology* 139 1395–1400. 10.1099/00221287-139-6-1395 8360630

[B18] de Vries R. P. Visser J. (2001). Aspergillus enzymes involved in degradation of plant cell wall polysaccharides. *Microbiol. Mole. Biol. Rev.* 65 497–522. 10.1128/MMBR.65.4.497-522.2001 PMC99039 11729262

[B19] Edwards J. E. Forster R. J. Callaghan T. M. Dollhofer V. Dagar S. S. Cheng Y. F. (2017). PCR and Omics based techniques to study the diversity, ecology and biology of anaerobic fungi: insights, challenges and opportunities. *Front. Microbiol.* 8:1657. 10.3389/fmicb.2017.01657 28993761 PMC5622200

[B20] Gruninger R. J. Puniya A. K. Callaghan T. M. Edwards J. E. Youssef N. Dagar S. S. (2014). Anaerobic fungi (phylum Neocallimastigomycota): advances in understanding their taxonomy, life cycle, ecology, role and biotechnological potential. *FEMS Microbiol. Ecology* 90 1–17. 10.1111/1574-6941.12383 25046344

[B21] Guerouali A. Wardeh M. F. (1998). “Assessing nutrient requirements and limits to production of the camel under its simulated natural environment,” in *Proceedings of the Third Annual Meeting for Animal Production under Arid Conditions*, (Al-Ain: United Arab Emirates), 36–51.

[B22] Hackstein J. H. P. Baker S. E. van Hellemond J. J. Tielens A. G. M. (2019). “Hydrogenosomes of anaerobic fungi: an alternative way to adapt to anaerobic environments,” in *Hydrogenosomes and Mitosomes: Mitochondria of Anaerobic Eukaryotes*, ed. Tachezy J. (Cham: Springer), 159–175.

[B23] Hanafy R. A. Lanjekar V. B. Dhakephalkar P. K. Callaghan T. M. Dagar S. S. Griffith G. W. (2020a). Seven new Neocallimastigomycota genera from wild, zoo-housed, and domesticated herbivores greatly expand the taxonomic diversity of the phylum. *Mycologia* 112 1212–1239. 10.1080/00275514.2019.1696619 32057282

[B24] Hanafy R. A. Johnson B. Youssef N. H. Elshahed M. S. (2020b). Assessing anaerobic gut fungal diversity in herbivores using D1/D2 large ribosomal subunit sequencing and multi-year isolation. *Environ. Microbiol.* 22 3883–3908. 10.1111/1462-2920.15164 32656919

[B25] Hanafy R. A. Youssef N. H. Elshahed M. S. (2021). Paucimyces polynucleatus gen. nov, sp. nov., a novel polycentric genus of anaerobic gut fungi from the faeces of a wild blackbuck antelope. *Int. J. Syst. Evol. Microbiol.* 71. 10.1099/ijsem.0.004832 34161217

[B26] He L. W. Zhou W. Xing Y. Q. Pian R. Q. Chen X. Y. Zhang Q. (2020). Improving the quality of rice straw silage with *moringa oleifera* leaves and propionic acid: fermentation, nutrition, aerobic stability and microbial communities. *Bioresou. Technol.* 299:122579. 10.1016/j.biortech.2019.122579 31855660

[B27] Hess M. Paul S. S. Puniya A. K. van der Giezen M. Shaw C. Edwards J. E. (2020). Anaerobic fungi: past, present, and future. *Front. Microbiol.* 11:584893. 10.3389/fmicb.2020.584893 33193229 PMC7609409

[B28] Hungate R. E. Smith W. Bauchop T. Yu I. Rabinowitz J. C. (1970). Formate as an intermediate in the bovine rumen fermentation. *J. Bacteriol.* 102 389–397. 10.1128/jb.102.2.389-397.1970 5419259 PMC247563

[B29] Jin W. Cheng Y. F. Mao S. Y. Zhu W. Y. (2011). Isolation of natural cultures of anaerobic fungi and indigenously associated methanogens from herbivores and their bioconversion of lignocellulosic materials to methane. *Bioresou. Technol.* 102 7925–7931. 10.1016/j.biortech.2011.06.026 21719276

[B30] Joshi A. Lanjekar V. B. Dhakephalkar P. K. Callaghan T. M. Griffith G. W. Dagar S. S. (2018). *Liebetanzomycespolymorphus* gen. et sp. nov., a new anaerobic fungus (Neocallimastigomycota) isolated from the rumen of a goat. *MycoKeys* 2018 89–110. 10.3897/mycokeys.40.28337 30364831 PMC6198248

[B31] Kazemi Shariat Panahi H. M. Dehhaghi G. J. Guillemin G. Gupta V. K. Lam S. S. Aghbashlo M. (2022). A comprehensive review on anaerobic fungi applications in biofuels production. *Sci. Total Environ.* 829:154521. 10.1016/j.scitotenv.2022.154521 35292323

[B32] Kumar S. Stecher G. Li M. Knyaz C. Tamura K. (2018). MEGA X: molecular evolutionary genetics analysis across computing platforms. *Mole. Biol. Evolu.* 35 1547–1549. 10.1093/molbev/msy096 29722887 PMC5967553

[B33] Li M. M. White R. R. Guan L. L. Harthan L. Hanigan M. D. (2021). Metatranscriptomic analyses reveal ruminal pH regulates fiber degradation and fermentation by shifting the microbial community and gene expression of carbohydrate-active enzymes. *Animal Microbiome* 3:32. 10.1186/s42523-021-00092-6 33892824 PMC8063335

[B34] Li Y. F. Jin W. Cheng Y. F. Zhu W. Y. (2016). Effect of the associated methanogen *Methanobrevibacter thaueri* on the dynamic profile of end and intermediate metabolites of anaerobic fungus *Piromyces* sp F1. *Curr. Microbiol.* 73 434–441. 10.1007/s00284-016-1078-9 27287262

[B35] Li Y. F. Jin W. Mu C. L. Cheng Y. F. Zhu W. Y. (2017). Indigenously associated methanogens intensified the metabolism in hydrogenosomes of anaerobic fungi with xylose as substrate. *J. Basic Microbiol.* 57 933–940. 10.1002/jobm.201700132 28791723

[B36] Li Y. Q. Hou Z. S. Shi Q. C. Cheng Y. F. Zhu W. Y. (2020). Methane production from different parts of corn stover via a simple co-culture of an anaerobic fungus and methanogen. *Front. Bioengineering Biotechnol.* 8:314. 10.3389/fbioe.2020.00314 32426337 PMC7204275

[B37] Li Y. Q. Meng Z. X. Xu Y. Shi Q. C. Ma Y. P. Aung M. (2021). Interactions between anaerobic fungi and methanogens in the rumen and their biotechnological potential in biogas production from lignocellulosic materials. *Microorganisms* 9:190. 10.3390/microorganisms9010190 33477342 PMC7830786

[B38] Li Y. Q. Xu Y. Xue Y. H. Yang S. H. Cheng Y. F. Zhu W. Y. (2022). Ethanol production from lignocellulosic biomass by co-fermentation with *Pecoramyces* sp. F1 and *Zymomonas mobilis* ATCC 31821 in an integrated process. *Biomass Bioenergy* 161:106454.

[B39] Liang J. S. Zhang H. B. Zhang P. Y. Zhang G. M. Cai Y. J. Wang Q. Y. (2021). Effect of substrate load on anaerobic fermentation of rice straw with rumen liquid as inoculum: hydrolysis and acidogenesis efficiency, enzymatic activities and rumen bacterial community structure. *Waste Manag.* 124 235–243. 10.1016/j.wasman.2021.02.017 33636425

[B40] Ma J. Zhong P. Li Y. Sun Z. Sun X. Aung M. (2022). Hydrogenosome, pairing anaerobic fungi and H(2)-utilizing microorganisms based on metabolic ties to facilitate biomass utilization. *J. Fungi* 8:338. 10.3390/jof8040338 35448569 PMC9026988

[B41] Mankar A. R. Pandey A. Modak A. Pant K. K. (2021). Pretreatment of lignocellulosic biomass: a review on recent advances. *Bioresou. Technol.* 334:125235. 10.1016/j.biortech.2021.125235 33957458

[B42] Mao S. Y. Huo W. J. Zhu W. Y. (2016). Microbiome-metabolome analysis reveals unhealthy alterations in the composition and metabolism of ruminal microbiota with increasing dietary grain in a goat model. *Environ. Microbiol.* 18 525–541. 10.1111/1462-2920.12724 25471302

[B43] Mizrahi I. Wallace R. J. Morais S. (2021). The rumen microbiome: balancing food security and environmental impacts. *Nat. Rev. Microbiol.* 19 553–566. 10.1038/s41579-021-00543-6 33981031

[B44] Nanda S. Azargohar R. Dalai A. K. Kozinski J. A. (2015). An assessment on the sustainability of lignocellulosic biomass for biorefining. *Renewable Sustainable Energy Rev.* 50 925–941.

[B45] Nguyen L. N. Nguyen A. Q. Johir M. A. Guo W. S. Ngo H. H. Chaves A. V. (2019). Application of rumen and anaerobic sludge microbes for bio harvesting from lignocellulosic biomass. *Chemosphere* 228 702–708. 10.1016/j.chemosphere.2019.04.159 31063917

[B46] Niu D. Z. Zuo S. S. Jiang D. Tian P. J. Zheng M. L. Xu C. C. (2018). Treatment using white rot fungi changed the chemical composition of wheat straw and enhanced digestion by rumen microbiota *in vitro*. *Animal Feed Sci. Technol.* 237 46–54. 10.1016/j.anifeedsci.2018.01.005

[B47] Oladosu Y. Mohd Y. R. Abdullah N. Magaji U. Ghazali Hussin Ramli A. (2016). Fermentation quality and additives: a case of rice straw silage. *BioMed. Res. Int.* 2016:7985167. 10.1155/2016/7985167 27429981 PMC4939334

[B48] Ozkose E. Thomas B. J. Davies D. R. Griffith G. W. Theodorou M. K. (2001). *Cyllamyces aberensis* gen.nov sp.nov., a new anaerobic gut fungus with branched sporangiophores isolated from cattle. *Canadian J. Bot. Revue Canadienne De Botanique* 79 666–673. 10.1139/b01-047

[B49] Rabee A. E. Forster R. J. Elekwachi C. O. Kewan K. Z. Sabra E. A. Shawket S. M. (2019). Community structure and fibrolytic activities of anaerobic rumen fungi in dromedary camels. *J. Basic Microbiol.* 59 101–110. 10.1002/jobm.201800323 30303547

[B50] Samsudin A. A. Evans P. N. Wright A. D. Al Jassim R. (2011). Molecular diversity of the foregut bacteria community in the dromedary camel (*Camelus dromedarius*). *Environ. Microbiol.* 13 3024–3035. 10.1111/j.1462-2920.2011.02579.x 21914099

[B51] Sawatdeenarunat C. Surendra K. C. Takara D. Oechsner H. Khanal S. K. (2015). Anaerobic digestion of lignocellulosic biomass: challenges and opportunities. *Bioresou. Technol.* 178 178–186. 10.1016/j.biortech.2014.09.103 25446783

[B52] Saye L. M. G. Navaratna T. A. Chong J. P. J. O’Malley M. A. Theodorou M. K. Reilly M. (2021). The anaerobic fungi: challenges and opportunities for industrial lignocellulosic biofuel production. *Microorganisms* 9:694. 10.3390/microorganisms9040694 33801700 PMC8065543

[B53] Shi Q. C. Li Y. Q. Li Y. F. Cheng Y. Zhu W. (2019). Effects of steam explosion on lignocellulosic degradation of, and methane production from, corn stover by a co-cultured anaerobic fungus and methanogen. *Bioresou. Technol.* 290:121796. 10.1016/j.biortech.2019.121796 31319215

[B54] Silva A. F. R. Brasil Y. L. Koch K. Amaral M. C. S. (2021). Resource recovery from sugarcane vinasse by anaerobic digestion-A review. *J. Environ. Manag.* 295:113137. 10.1016/j.jenvman.2021.113137 34198179

[B55] Singh J. Suhag M. Dhaka A. (2015). Augmented digestion of lignocellulose by steam explosion, acid and alkaline pretreatment methods: a review. *Carbohydrate Polymers* 117 624–631. 10.1016/j.carbpol.2014.10.012 25498680

[B56] Solomon K. V. Haitjema C. H. Henske J. K. Gilmore S. P. Borges-Rivera D. Lipzen A. (2016). Early-branching gut fungi possess a large, comprehensive array of biomass-degrading enzymes. *Science* 351 1192–1195. 10.1126/science.aad1431 26912365 PMC5098331

[B57] Stabel M. Hanafy R. A. Schweitzer T. Greif M. Aliyu H. Flad V. (2020). *Aestipascuomyces dupliciliberans* gen. nov, sp. nov., the first cultured representative of the uncultured SK4 clade from Aoudad sheep and alpaca. *Microorganisms* 8:1734. 10.3390/microorganisms8111734 33167420 PMC7694369

[B58] Stevenson D. M. Weimer P. J. (2002). Isolation and characterization of a Trichoderma strain capable of fermenting cellulose to ethanol. *Appl. Microbiol. Biotechnol.* 59 721–726. 10.1007/s00253-002-1027-3 12226731

[B59] Sugiharto S. Yudiarti T. Isroli I. (2016). Performances and haematological profile of broilers fed fermented dried cassava (Manihot esculenta Crantz). *Tropical Animal Health Production* 48 1337–1341. 10.1007/s11250-016-1098-2 27307279

[B60] Taherzadeh M. J. Karimi K. (2008). Pretreatment of lignocellulosic wastes to improve ethanol and biogas production: a review. *Int. J. Mole. Sci.* 9 1621–1651. 10.3390/ijms9091621 19325822 PMC2635757

[B61] Theodorou M. K. Williams B. A. Dhanoa M. S. Mcallan A. B. France J. (1994). A simple gas-production method using a pressure transducer to determine the fermentation kinetics of ruminant feeds. *Animal Feed Sci. Technol.* 48 185–197. 10.1016/0377-8401(94)90171-6

[B62] Trinci A. P. J. Davies D. R. Gull K. Lawrence M. I. Nielsen B. B. Rickers A. (1994). Anaerobic fungi in herbivorous animals. *Mycological Res.* 98 129–152. 10.1016/S0953-7562(09)80178-0

[B63] Vansoest P. J. Robertson J. B. Lewis B. A. (1991). Methods for dietary fiber, neutral detergent fiber, and nonstarch polysaccharides in relation to animal nutrition. *J. Dairy Sci.* 74 3583–3597. 10.3168/jds.S0022-0302(91)78551-2 1660498

[B64] Voigt K. James T. Y. Kirk P. M. Santiago A. L. C. M. D. Waldman B. Griffith G. W. (2021). Early-diverging fungal phyla: taxonomy, species concept, ecology, distribution, anthropogenic impact, and novel phylogenetic proposals. *Fungal Diversity* 109 59–98. 10.1007/s13225-021-00480-y 34608378 PMC8480134

[B65] Wilken S. E. Monk J. M. Leggieri P. A. Lawson C. E. Lankiewicz T. S. Seppala S. (2021). Experimentally validated reconstruction and analysis of a genome-scale metabolic model of an anaerobic Neocallimastigomycota fungus. *mSystems* 6 e00002–e21. 10.1128/mSystems.00002-21 33594000 PMC8561657

[B66] Wilson R. T. (1989). *The Nutritional Requirements of Camel.* Ouargla: CIHEAM-IAMZ.

[B67] Yamamoto M. Iakovlev M. Bankar S. Tunc M. S. van Heiningen A. (2014). Enzymatic hydrolysis of hardwood and softwood harvest residue fibers released by sulfur dioxide-ethanol-water fractionation. *Bioresou. Technol.* 167 530–538. 10.1016/j.biortech.2014.06.054 25022728

[B68] Yang X. X. Chen H. Z. Gao H. L. Li Z. H. (2001). Bioconversion of corn straw by coupling ensiling and solid-state fermentation. *Bioresou. Technol.* 78 277–280. 10.1016/s0960-8524(01)00024-4 11341688

[B69] Zabed H. Sahu J. N. Boyce A. N. Faruq G. (2016). Fuel ethanol production from lignocellulosic biomass: an overview on feedstocks and technological approaches. *Renewable Sustainable Energy Rev.* 66 751–774.

[B70] Zhu W. Y. Mao S. Y. Wang Q. J. Yao W. Theodorou M. K. (2001). Study on the screening of anaerobic fungi by *in vitro* fermentation. *J. Nanjing Agricultural University* 24 44–48.

