# Supplementary material for: Anaerobic Fungi Isolated From Bactrian Camel Rumen Contents Have Strong Lignocellulosic Bioconversion Potential
Source: Front Microbiol. 2022 Jul 19;13:888964. doi: 10.3389/fmicb.2022.888964 (PMC9345502; doi:10.3389/fmicb.2022.888964)
Supplement: Supplementary file 1 [file Data_Sheet_1.docx]

**Supplementary Information**

**Supplementary Table 1. Characterization of anaerobic fungal strains from the isolation.**

| Cultures | Host | Morphology | Genera |
| --- | --- | --- | --- |
| CR1 -21 | Bactrian camel rumen contents | Monocentric, Uniflagellate, Filamentous | *Oontomyces* |
| CF1 -10 | Bactrian camel feces | Monocentric, Uniflagellate, Filamentous | *Piromyces* |

**Supplementary Table 2. Fermentation results of fungus obtained from rumen contents on rice straw. Data were showed as mean ± SEM (n=4) and ^a, b, c,….^, means with different letters in the same column are different (*P* < 0.05).**

| Cultures | Total Gas Production (mL) | DM Loss (%) | Per DM Gas Production (mL/g) | pH Value |
| --- | --- | --- | --- | --- |
| CR1 | 140.9 ± 2.6^cdefgh^ | 37.23 ± 0.83 ^abc^ | 403.15 ± 14.71^bcd^ | 6.38 ± 0.01 ^ghi^ |
| CR2 | 158.1 ± 1.9^a^ | 39.69 ± 0.69 ^ab^ | 424.08 ± 12.12 ^abcd^ | 6.49 ± 0.03 ^bcdefg^ |
| CR3 | 149.6 ± 10.6^abcde^ | 37.57 ± 0.47 ^abc^ | 423.70 ± 30.42 ^abcd^ | 6.50 ± 0.02 ^bcdef^ |
| CR4 | 135.9 ± 1.9^fgh^ | 32.00 ± 0.67 ^c^ | 451.99 ± 3.26 ^abc^ | 6.43 ± 0.08 ^efgh^ |
| CR5 | 144.6 ± 2.4^bcdefg^ | 32.36 ± 2.83 ^c^ | 483.74 ± 48.01 ^ab^ | 6.39 ± 0.01 ^fghi^ |
| CR6 | 143.9 ± 1.4^bcdefg^ | 33.12 ± 3.02 ^c^ | 469.91 ± 43.24 ^ab^ | 6.36 ± 0.04 ^hi^ |
| CR7 | 149.6 ± 2.8^abcde^ | 39.06 ± 1.24 ^ab^ | 407.80 ± 5.90 ^abcd^ | 6.57 ± 0.03 ^abc^ |
| CR8 | 151.8 ± 1.2^abc^ | 33.63 ± 1.02 ^c^ | 480.86 ± 12.55 ^ab^ | 6.59 ± 0.03 ^ab^ |
| CR9 | 150.2 ± 3.5^abcd^ | 32.82 ± 1.65 ^c^ | 490.27 ± 32.82 ^a^ | 6.49 ± 0.05 ^bcdefg^ |
| CR10 | 145.6 ± 1.6^abcdefg^ | 32.84 ± 0.77 ^c^ | 472.34 ± 15.16 ^ab^ | 6.23 ± 0.02 ^j^ |
| CR11 | 154.6 ± 1.3^ab^ | 36.76 ± 2.39 ^abc^ | 451.22 ± 30.61 ^abc^ | 6.48 ± 0.05 ^bcdefg^ |
| CR12 | 142.1 ± 10.1 ^bcdefg^ | 40.04 ± 0.44 ^ab^ | 377.66 ± 26.99 ^cd^ | 6.49 ± 0.08 ^bcdefg^ |
| CR13 | 133.8 ± 2.4 ^gh^ | 39.73 ± 0.78 ^ab^ | 358.77 ± 12.23 ^d^ | 6.55 ± 0.03 ^abcd^ |
| CR14 | 129.1 ± 1.8 ^h^ | 32.04 ± 0.58 ^c^ | 428.90 ± 1.94 ^abcd^ | 6.56 ± 0.02 ^abc^ |
| CR15 | 137.4 ± 2.3 ^defgh^ | 32.34 ± 2.45 ^c^ | 458.00 ± 40.55 ^abc^ | 6.44 ± 0.01 ^defgh^ |
| CR16 | 142.1 ± 2.7 ^bcdefg^ | 41.45 ± 1.17^a^ | 364.95 ± 4.08 ^d^ | 6.45 ± 0.01 ^cdefgh^ |
| CR17 | 136.7 ± 1.3 ^efgh^ | 35.86 ± 2.84 ^bc^ | 410.49 ± 32.17 ^abcd^ | 6.41 ± 0.05 ^fgh^ |
| CR18 | 144.2 ± 1.1 ^bcdefg^ | 36.33 ± 0.96 ^abc^ | 422.65 ± 9.38 ^abcd^ | 6.63 ± 0.03 ^a^ |
| CR19 | 146.8 ± 1.2 ^abcdef^ | 39.29 ± 2.25 ^ab^ | 400.34 ± 24.22 ^bcd^ | 6.29 ± 0.02 ^ij^ |
| CR20 | 138.3 ± 1.5 ^defgh^ | 32.75 ± 0.66 ^c^ | 449.85 ± 13.08 ^abc^ | 6.55 ± 0.05 ^abcde^ |
| CR21 | 142.7 ± 3.3 ^bcdefg^ | 35.57 ± 1.56 ^bc^ | 429.02 ± 26.07 ^abcd^ | 6.65 ± 0.03^a^ |
| *P* value | < 0.05 | < 0.05 | < 0.05 | < 0.05 |

**Supplementary Table 3. Fermentation results of fungus obtained from feces on rice straw. Data were showed as mean ± SEM (n=4) and ^a, b, c,….^, means with different letters in the same column are different (*P* < 0.05).**

| Cultures | Total Gas Production (mL) | DM Loss (%) | Per DM Gas Production (mL/g) | pH Value |
| --- | --- | --- | --- | --- |
| CF1 | 122.3 ± 2.1^b^ | 37.37 ± 1.53 | 347.6 ± 8.5^ab^ | 6.37 ± 0.04^a^ |
| CF2 | 118.5 ± 2.6^b^ | 34.75 ± 4.29 | 375.6 ± 57.2^ab^ | 6.36 ± 0.02^ab^ |
| CF3 | 122.8 ± 2.0^b^ | 38.19 ± 2.15 | 343.2 ± 20.4^ab^ | 6.30 ± 0.05^abc^ |
| CF4 | 119.8 ± 0.2^b^ | 40.46 ± 0.76 | 314.2 ± 5.5^a^ | 6.35 ± 0.03^ab^ |
| CF5 | 118.9 ± 1.9^b^ | 40.13 ± 1.76 | 315.6 ± 17.6^a^ | 6.34 ± 0.04^abc^ |
| CF6 | 105.0 ± 1.7^c^ | 37.35 ± 2.32 | 301.0 ± 22.3^a^ | 6.28 ± 0.02^abc^ |
| CF7 | 124.8 ± 3.5^b^ | 39.32 ± 1.22 | 337.0 ± 11.2^ab^ | 6.24 ± 0.00^c^ |
| CF8 | 119.5 ± 2.3^b^ | 40.12 ± 2.61 | 319.2 ± 25.3^a^ | 6.26 ± 0.04^bc^ |
| CF9 | 136.2 ± 1.9^a^ | 37.95 ± 1.18 | 381.2 ± 12.0^ab^ | 6.29 ± 0.02^abc^ |
| CF10 | 135.6 ± 0.7^a^ | 34.72 ± 2.21 | 417.7 ± 28.5^a^ | 6.28 ± 0.02^abc^ |
| *P* value | < 0.05 | < 0.05 | < 0.05 | < 0.05 |

**Supplementary Figures**

 **Figure S1. Cumulative gas production from the fungus from rumen contents (A) and feces (B).**

**Figure S2. Activities of fiber-degrading enzymes (A: CMCase; B: Xylanase) of cultures using different substrates (R: Reed; B: Broussonetia papyrifera leaves; A: Alfalfa stalk; M: Melilotus officinalis). These error bars represent the standard error of the mean (n = 4**) **and ^a, b, c^, means with different letters in the same column are different (*P* < 0.05).**
